# Mechanisms of delayed ischemia/reperfusion evoked ROS generation in the hippocampal CA1 zone of adult mouse brain slices

**DOI:** 10.1038/s41598-025-07070-x

**Published:** 2025-07-02

**Authors:** Yuliya V. Medvedeva, Edward Sharman, John H. Weiss

**Affiliations:** 1 Department of Neurology, University of California, Irvine, Irvine, CA 92697-4299 USA; 2 Department of Anatomy and Neurobiology, University of California, Irvine, Irvine, CA 92697-4299 USA

**Keywords:** Hippocampal slice, Mitochondria, Mitochondrial hyperpolarization, Zn^2+^, MCU, Oxygen glucose deprivation

## Abstract

ROS overproduction is an important contributor to delayed ischemia/reperfusion induced neuronal injury, but relevant mechanisms remain poorly understood. We used oxygen–glucose deprivation (OGD)/reperfusion in mouse hippocampal slices to investigate ROS production in the CA1 pyramidal cell layer during and after transient ischemia. OGD evoked a 2-stage increase in ROS production: 1st—an abrupt increase in ROS generation starting during OGD followed by a marked slowing; and 2nd—a sharp ROS burst starting ~ 40 min after reperfusion. We further found that a slight mitochondrial hyperpolarization occurs shortly after OGD termination. Consequently, we showed that administration of low dose FCCP or of FTY720 (both of which cause mild, ~ 10%, mitochondrial depolarization), markedly diminished the delayed ROS burst, suggesting that mitochondrial hyperpolarization contributes to ROS production after reperfusion. Zn^2+^ chelation after OGD withdrawal also substantially decreased the late surge of ROS generation—in line with our prior studies indicating a critical contribution of Zn^2+^ entry into mitochondria via the mitochondrial Ca^2+^ uniporter (MCU) to mitochondrial damage after OGD. Thus, reperfusion-induced mitochondria hyperpolarization and mitochondrial Zn^2+^ accumulation both contribute to mitochondrial ROS overproduction after ischemia. As these events occur after reperfusion, they may be amenable to therapeutic interventions.

## Introduction

Brain ischemia is a leading cause of disability and mortality in the aging population. After brief ischemic episodes, many vulnerable neurons like hippocampal pyramidal neurons often initially recover but die hours to days later^1,2^. Considerable evidence supports the view that oxidative stress due to ROS overproduction is a key contributor to the delayed neuronal injury, damaging tissue and contributing to activation of cell death pathways^3,4^. Consequently, it was suggested that substantial amounts of ROS are produced after restoration of blood flow (reperfusion), and thus oxygen and glucose supply. Unfortunately, antioxidants have shown little benefits in clinical trials, likely due to inadequate access to the sites of damage. Therefore, elucidating the mechanisms underlying ROS overproduction is essential for identifying new therapeutic targets.

Information currently available about the dynamics and mechanisms of post-ischemic ROS generation is insufficient for the development of therapeutic approaches. While several possible contributors have been proposed including mitochondria, NADPH oxidase (NOX) and xanthine oxidase^5–7^, mitochondria are believed to be the dominant source responsible for 80–90% of post-ischemic ROS production^8,9^.

Neuronal Ca^2+^ overload has long been considered a major player in ischemic ROS production^5,10,11^ however the mechanism was not clearly understood. A number of studies investigating Ca^2+^ effect on mitochondria laid the base for a recently proposed hypothesis highlighting Ca^2+^-mediated mitochondrial hyperpolarization as a key contributor to reperfusion-associated ROS overproduction^9^. Specifically, Ca^2+^ accumulates in neurons during ischemia and quickly enters mitochondria through the mitochondrial Ca^2+^ uniporter (MCU)^12–14^. Under physiological conditions of increased energy demand, elevated mitochondrial Ca^2+^ accumulation increases activity of multiple mitochondrial proteins involved in oxidative phosphorylation consequently leading to transient moderate increases in mitochondrial respiration and ROS production^9,15–17^. However, ischemic conditions, that lead to strong cytoplasmic and, thus, mitochondrial Ca^2+^ overload likely result in considerable overactivation of the respiratory proteins, and probably lead to a hyperactive state of the electron transport chain (ETC) upon reintroduction of oxygen^9^. This, in turn, likely results in prolonged mitochondrial hyperpolarization and a burst of ROS generation, as several studies have found that mitochondrial hyperpolarization (when the mitochondrial membrane potential, ΔΨ_m_, is more negative than ~  − 140 mV) causes an exponential increase in ROS production^18,19^.

Another factor likely contributing to ischemic ROS overproduction is mitochondrial Zn^2+^ accumulation. Zn^2+^ accumulates in selectively vulnerable neurons after ischemia^20,21^, and enters mitochondria via the MCU where it stays for prolonged periods and contributes to mitochondrial dysfunction^22–26^. Multiple studies using cell culture have shown that Zn^2+^ accumulation in mitochondria is a highly potent trigger of ROS generation^27–31^.

Our recent studies using an ischemia model in brain slices have shown that both Ca^2+^ and Zn^2+^ accumulate in mitochondria of hippocampal CA1 neurons during ischemia; next, when mitochondria depolarize due to insufficient supply of ATP, these ions are released back to the cytoplasm; finally, upon reperfusion and recovery of ΔΨ_m_, both Ca^2+^ and Zn^2+^ re-enter mitochondria. Ca^2+^ seem to be cleared from mitochondria relatively quickly, but Zn^2+^ stays there for a prolonged period (more than 1 h)^24–26^. We suggest that the persistent mitochondrial Zn^2+^ accumulation, which occurs in CA1 neurons after ischemia/reperfusion, is an important contributor to ROS overproduction.

Our study uses an ex vivo oxygen/glucose deprivation (OGD) model of ischemia/reperfusion in acute brain slices loaded with a ROS sensitive indicator to assess the dynamics of ROS generation during and after short ischemia in the CA1 hippocampal region. We observed a sharp increase in ROS generation during and for several minutes after the ischemic episode. However, after this burst, ROS production considerably subsides for 20–30 min, before the occurrence of a second delayed sharp surge in ROS generation. Next, we utilized pharmacological interventions delivered after the ischemic episode to examine mechanisms contributing to the surge in ROS generation after reperfusion. We found that delayed mitochondrial hyperpolarization, and Zn^2+^ uptake to mitochondria through the MCU are both major contributors to post-ischemic ROS overproduction. Interventions targeting these mechanisms can be delivered after restoration of blood flow and, thus, might represent promising therapeutic approaches to decrease delayed brain injury after ischemia.

## Materials and methods

### Animals

All studies were performed according to the experimental protocols approved by the University of California Irvine Animal Care and Use Committee (IACUC). All methods were carried out in accordance with relevant guidelines and recommendations of the IACUC. All methods of euthanasia were carried out in accordance with the ​American Veterinary Medical Association​ (AVMA) guidelines. All studies were performed and reported in accordance with the ARRIVE guidelines. Efforts were made to minimize animal numbers and suffering. Both male and female mice from 129S6/SvEvTac strain (Taconic Biosciences) were utilized for preparation of brain slices.

### Preparation of acute hippocampal slices

Slices were prepared from the brains of ~ 4 week old mice (both sexes) as previously described (Medvedeva et al., 2009). Mice were deeply anesthetized with isoflurane and decapitated. The brains were quickly removed and placed in an ice-cold solution containing (in mM): 220 sucrose, 3 KCl, 1.25 NaH_2_PO_4_, 6 MgSO_4_, 26 NaHCO_3_, 0.2 CaCl_2_, 10 glucose and 0.42 ketamine (pH 7.35, 310 mOsm, equilibrated with 95% O2 / 5% CO2). Horizontal brain slices (300 µm) were cut using a Leica VT1200 vibratome (Leica, Germany) which has close to zero vertical vibration, thereby minimizing damage to the neurons caused by cutting. Slices were placed in artificial cerebro-spinal fluid (ACSF) containing (in mM): 126 NaCl, 3 KCl, 1.25 NaH_2_PO_4_,1 MgSO_4_, 26 NaHCO_3_, 2 CaCl_2_, 10 Glucose (pH 7.35, 310 mOsm, equilibrated with 95% O2 / 5% CO2). Slices were incubated for 1 h at 34 ± 0.5ºC, and then kept at room temperature (RT, 20-23ºC) in oxygenated ACSF.

### Fluorescent measurements

All recordings were obtained from the pyramidal neuron rich CA1 cell layer of the hippocampus in slices placed on the stage of an Olympus BX51WI microscope (Olympus, Japan) using a 40X objective. Experiments were performed at 32 ± 0.5ºC with a flow speed 2 ml/min. Recordings were carried out from the cell layer 30–50 µm below the slice surface. To investigate the dynamic changes in ROS production, slices were bath loaded with the ROS-sensitive indicator Hydroethidine (HEt, 10 µM, at RT)^24,32^. HEt was excited at 540(25) nm and emitted fluorescence was collected at 605(55) nm. To assess changes in ∆Ψ_m_, slices were bath loaded with Rhodamine 123 (Rhod123, 26 µM, 30 min at RT). Rhod123 is positively charged and accumulates in negatively charged mitochondria where its fluorescence is quenched. Upon loss of ∆Ψ_m_ the indicator is released into the cytoplasm causing an increase in fluorescence^33^. Rhod123 was excited at 540(25) nm and emitted fluorescence was collected at 605(55) nm. Carbonyl cyanide-4-(trifluoromethoxy)phenylhydrazone (FCCP, 2 µM) was applied at the indicated time points to evoke full loss of ∆Ψ_m_ and consequent maximal increase in Rhod123 fluorescence, providing a measure of the level of ∆Ψ_m_ before FCCP application. HEt and Rhod123 images were acquired every 15 s using MetaFluor imaging software (Molecular Devices, San Jose, CA). Data are presented as ΔF = (F-F_0_)/F_0_ where F is the current fluorescence intensity and F_0_ is the baseline fluorescence intensity.

### OGD/reperfusion model in slices and drug administration

To induce ischemia-like conditions in brain slices, ACSF was changed to an identical solution but without glucose (it was substituted with equimolar sucrose to maintain osmolarity) and bubbled with 95% N_2_/5% CO_2_. OGD was terminated after 6 min by restoration of oxygenated ACSF containing glucose, as we found that longer episodes of OGD caused substantial neuronal swelling evoked by spreading depolarization events usually occurring between 6 and 8 min after the start of OGD. This short OGD evoked a substantial burst in ROS production while causing minimal cell swelling.

Experimental drugs [the Zn^2+^ chelator TPEN (N,N,N',N'-tetrakis(2-pyridylmethyl)ethane-1,2-diamine, 20 µM), the MCU inhibitor Ru265 (5 µM), the NOX inhibitor apocynin (500 µM), FTY720 (10 µM), and low-dose FCCP (100 nM)] were applied 0–5 min after OGD withdrawal as indicated. FCCP (2 µM) was applied at the end of some experiments to evoke complete loss of ∆Ψ_m_.

### Reagents

Hydroethidine (Dihydroethidium) and FCCP (Carbonyl cyanide 4-(trifluoromethoxy)phenylhydrazone) were purchased from Cayman Chemical (Ann Arbor, MI). FTY720 (fingolimod) was obtained from APExBIO (Boston, MA). Rhodamine123 was obtained from Invitrogen (Carlsbad, CA). TPEN was purchased from Tocris (Bristol, UK), and ketamine was obtained from Sigma (St. Louis, MO). Ru265 was a gift from J. Wilson (Cornell University, Ithaca, NY). All other reagents were purchased from ThermoFisher Scientific (Waltham, MA).

### Experimental design and statistical analyses

**Fig. 1:**
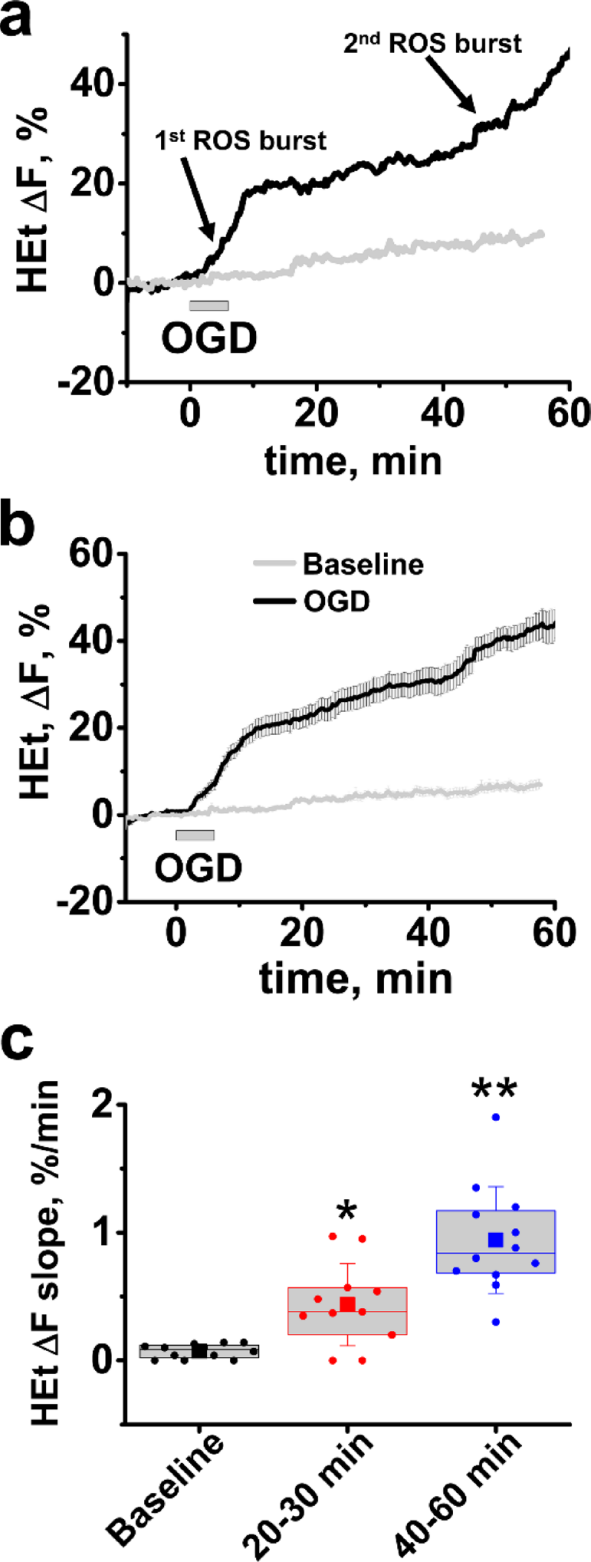
OGD/reperfusion evokes a two-phase acceleration in ROS production. Slices were bath loaded with HEt and subjected to OGD/reperfusion. Traces demonstrate dynamic changes in HEt fluorescence in the CA1 zone of hippocampus during OGD/reperfusion (black traces) and in slices not subjected to treatment (gray traces) in a representative slice (**a**) and as an average (± SE) of 14 slices (**b**). (**c**) Box chart represents the average rate of HEt ΔF changes (with borders at the 25th and 75th percentile, presented as % per min) during baseline, and during 2 time intervals—from 20–30 min, and from 40–60 min after OGD withdrawal. Circle symbols show data points for individual slices, squares show mean value, central line demonstrate median, and error bars show SD of mean. * *p* < 0.019 vs baseline, ** *p* < 0.01 compared to both baseline and 20–30 min intervals.

Statistical differences were assessed using one-Way ANOVA Tukey test in most of our experiments comprising 3 or more groups of data. In some experiments (when specified in Results section) we used a two-sample t-Test for comparison between two groups. To assess the rate of ROS generation after OGD (Fig. 1C) we evaluated the speed of HEt fluorescence changes by linear fitting of specific areas on the trace (baseline, 20–30 min and 40–60 min intervals after OGD withdrawal). Data were analyzed using Origin software (OriginLab, Northampton, MA). Our preliminary experiments did not find differences of investigated parameters between slices of female and male animals. In particular, we observed a 2-phase increase in ROS generation in animals of both sexes, and found no differences in HEt fluorescence levels at 50 min after the start of OGD between male and female groups (n = 6 males and 9 females, *p* = 0.97, two-sample t-Test). Thus, all statistical analyses included slices from mice of both sexes. All comparisons reflect sets of data substantially interleaved in time and were based on 7–20 slices from ≥ 6 animals for each condition (numbers of slices are indicated for each experiment).

## Results

### Ischemia/reperfusion evokes a 2-stage acceleration in ROS production

Our past studies observed that ROS generation is considerably increased during ischemia^24^. The current study focuses on events occurring after reperfusion—the period most amenable to interventions. As possibilities for dynamic ROS monitoring are very limited in animal models, we used ex vivo slices from adult mice. Slices were loaded with the ROS-sensitive indicator Hydroethidine (HEt) and subjected to short (6 min) OGD followed by reperfusion. Fluorescent measurements were obtained from the CA1 region of hippocampus. We found that OGD/reperfusion causes a pronounced 2-stage acceleration in ROS generation: the first one begins 2–3 min after the start of OGD, lasts for ~ 10 min, and is followed by a marked slowdown; the second stage starts ~ 40 min after OGD withdrawal (Fig. 1a,b) (rate of HEt ΔF changes was 0.07 ± 0.01%/min at baseline, 0.43 ± 0.09%/min at 20–30 min interval and 0.94 ± 0.12%/min between 40 and 60 min, n = 12, Fig. 1c).

### Mitochondrial hyperpolarization after OGD

As discussed in the introduction, we hypothesize that OGD/reperfusion evoked Ca^2+^ accumulation in mitochondria causes mitochondrial hyperpolarization after reperfusion, and a resulting remarkable increase in ROS production. We started testing our hypothesis by assessing whether mitochondrial hyperpolarization indeed occurs after OGD. To evaluate ∆Ψ_m_ we loaded slices with the fluorescent indicator, Rhodamine 123 (Rhod123) as in our previous studies^25,26^. This positively charged indicator is sequestered in negatively charged mitochondria, where its fluorescence is quenched, and the accumulated amount is proportional to ∆Ψ_m_. After partial or full ∆Ψ_m_ loss, Rhod123 is released to the cytoplasm. Treatment with the protonophore FCCP (2 μM) dissipates the proton gradient across the mitochondrial inner membrane and evokes full ∆Ψ_m_ dissipation, leading to Rhod123 release into the cytosol and a corresponding increase in fluorescence (∆F).

**Fig. 2:**
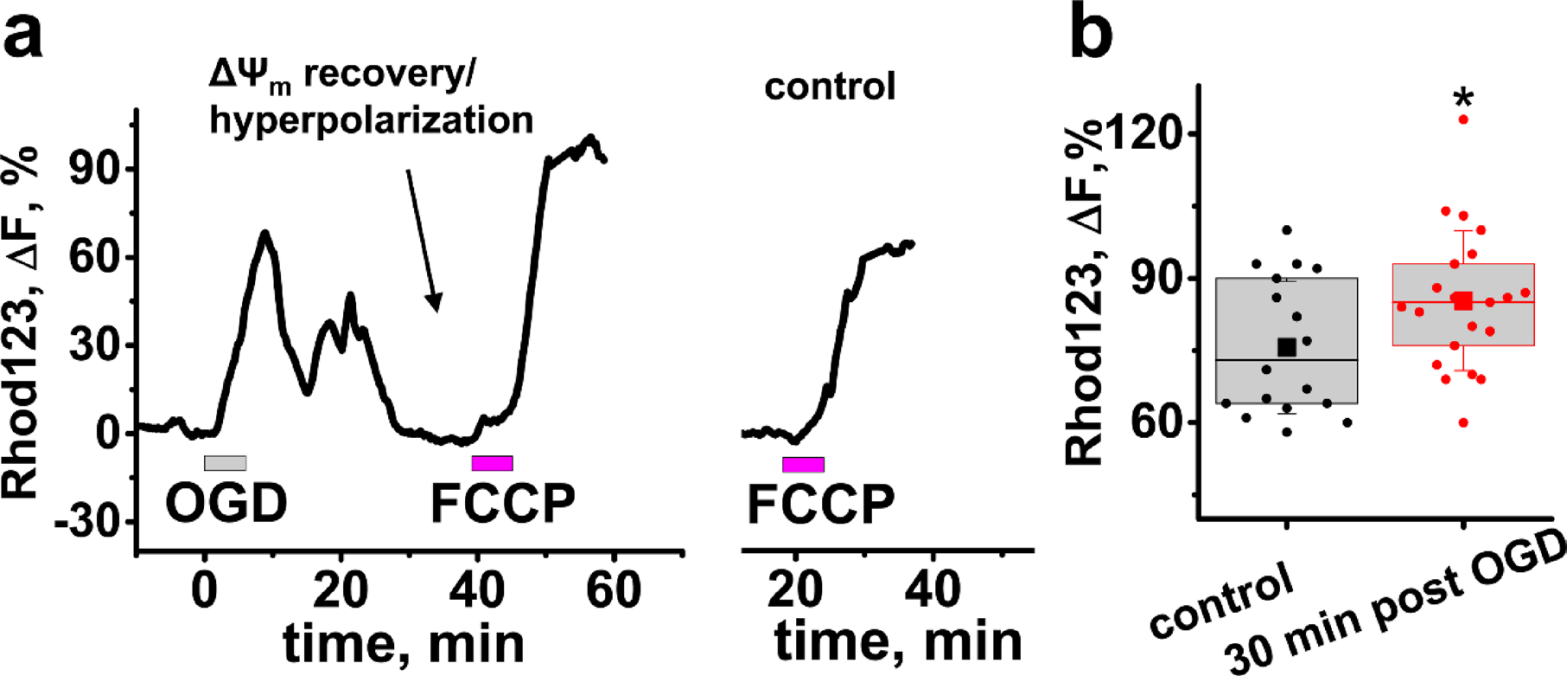
Mitochondria are moderately hyperpolarized 30 min after OGD withdrawal. Slices were loaded with Rhod123. (**a**) Representative traces show changes of Rhod123 fluorescence in the CA1 pyramidal cell zone of a slice exposed to OGD followed 30 min later by FCCP (2 μM, Left), or in a control slice treated with FCCP only (Right). (**b**) Box chart represents Rhod123 fluorescence changes in responce to FCCP treatment (these changes are indicative of the ∆Ψ_m_ prior to FCCP exposure) in slices ~ 30 min after an OGD episode or in control untreated slices. The squire symbol point to average, borders show 25th and 75th percentiles, circle symbols show data points for individual slices, and error bars indicate standard deviation. , *p = 0.038 vs control.

This ∆F is indicative of the ∆Ψ_m_ prior to FCCP exposure. After subjecting brain slices to the short (6 min) OGD we observed the ∆Ψ_m_ loss followed by complete recovery 10–30 min after OGD withdrawal (Fig. 2a). 30 min after the OGD termination we applied FCCP (2 μM) and found that the resulting increase in Rhod123 ∆F slightly but significantly exceeded that obtained from slices not subjected to OGD (control), indicating hyperpolarization of ∆Ψ_m_ after ischemia (Fig. 2b, Rhod123 ΔF = 85.3 ± 3.2%, n = 21 after ischemia vs 75.3 ± 3.8% in control, n = 18, *p* = 0.038, two-sample t-Test, Fig. 2).

### Mild mitochondrial depolarization with low dose FCCP or FTY720 considerably decreases ROS generation after ischemia

**Fig. 3:**
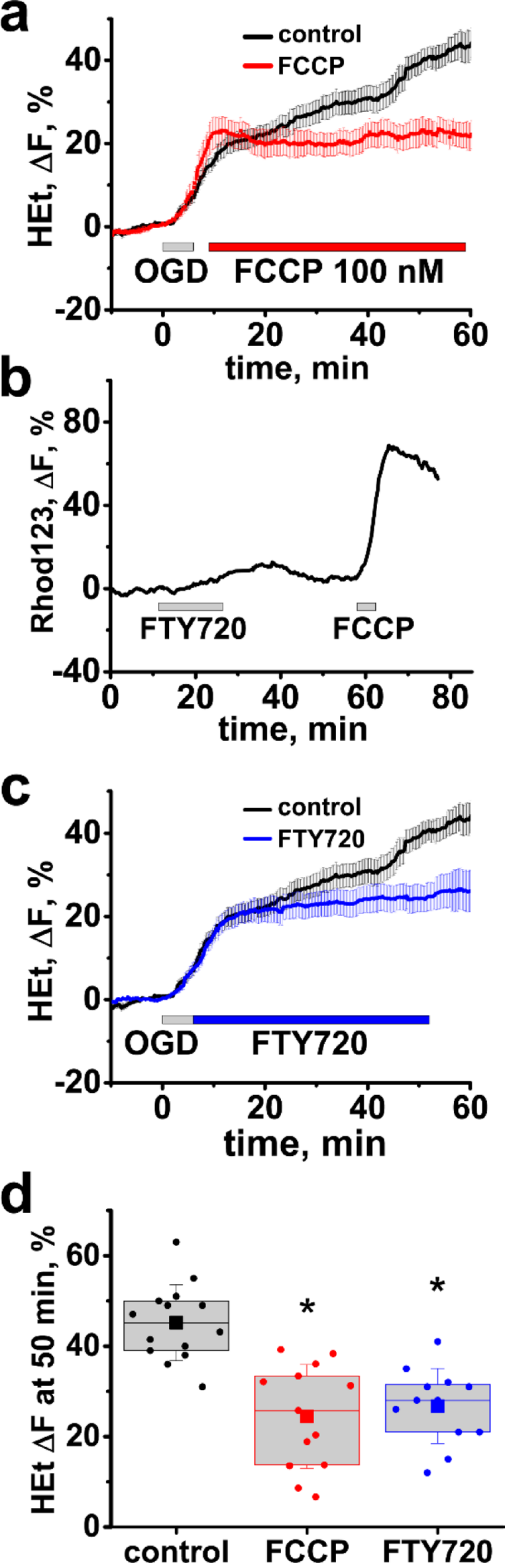
Mild mitochondrial depolarization inhibits the reperfusion evoked ROS burst in CA1. (**a**) Low dose FCCP (100 nM) decreases ROS generation after OGD. Slices were bath loaded with HEt and subjected to short OGD followed by application of FCCP 5 min later. Traces show average (± SE) changes in HEt ΔF in control slices subjected to OGD alone (black) and in slices treated with FCCP (red). (**b**) FTY720 causes a slight and reversible loss of ∆Ψ_m_. Slices were bath loaded with Rhod123. Representative trace (from n = 11) show changes in Rhod123 fluorescence after application of FTY720 (10 µM) for 15 min followed by FCCP (2 µM) 30 min later, as indicated. (**c**) FTY720 inhibits the reperfusion-evoked ROS generation. Traces show average changes in HEt fluorescence in control slices (black) and in slices treated withFTY720 (blue, 10 µM) after OGD termination, as indicated. d Box chart shows average (square symbol, borders are at 25th and 75th percentiles) HEt ΔF 50 min after the start of the OGD episode in control slices and in slices treated with FCCP or FTY720. Circle symbols show data points for individual slices, and error bars indicate standard deviation. *p < 0.01 vs control.

Next, we tested whether a slight ∆Ψ_m_ dissipation after reperfusion can mitigate the ROS overproduction. We observed that a low dose of the protonophore FCCP (100 nM) evokes a slight increase in Rhod123 fluorescence (8.6 ± 0.8%, n = 8, data not shown) in the CA1 hippocampal zone indicative of a mild loss of ∆Ψ_m_. Subsequently, we found that applying FCCP (100 nM, 5 min after OGD withdrawal for 50 min) considerably decreased the reperfusion-evoked ROS overproduction (HEt ΔF = 24.4 ± 3.2%, n = 13 slices, vs 39.7 ± 2.8%, n = 14 in control (OGD alone), *p* < 0.01, assessed 50 min after OGD onset, Fig. 3a,d).

As FCCP is not suitable as a drug, we searched for another agent capable of mildly depolarizing mitochondria. FTY720 (an FDA approved drug for treatment of multiple sclerosis) was shown to evoke mitochondrial depolarization in cultured cells^34^. In brain slices, we found that 10 μM of FTY720 causes a small and reversible mitochondrial depolarization in the hippocampal CA1 zone (9.40 ± 0.7%, n = 11, Fig. 3b). Next, we found that an exposure to FTY720 after OGD termination substantially inhibited the ROS burst after reperfusion (HEt ΔF = 25.1 ± 3.0%, n = 12 slices, *p* < 0.01 vs control, evaluated 50 min after OGD onset, Fig. 3c,d).

### Mitochondrial Zn^2+^ accumulation through the MCU after ischemia contributes to post-ischemic ROS overproduction

**Fig. 4:**
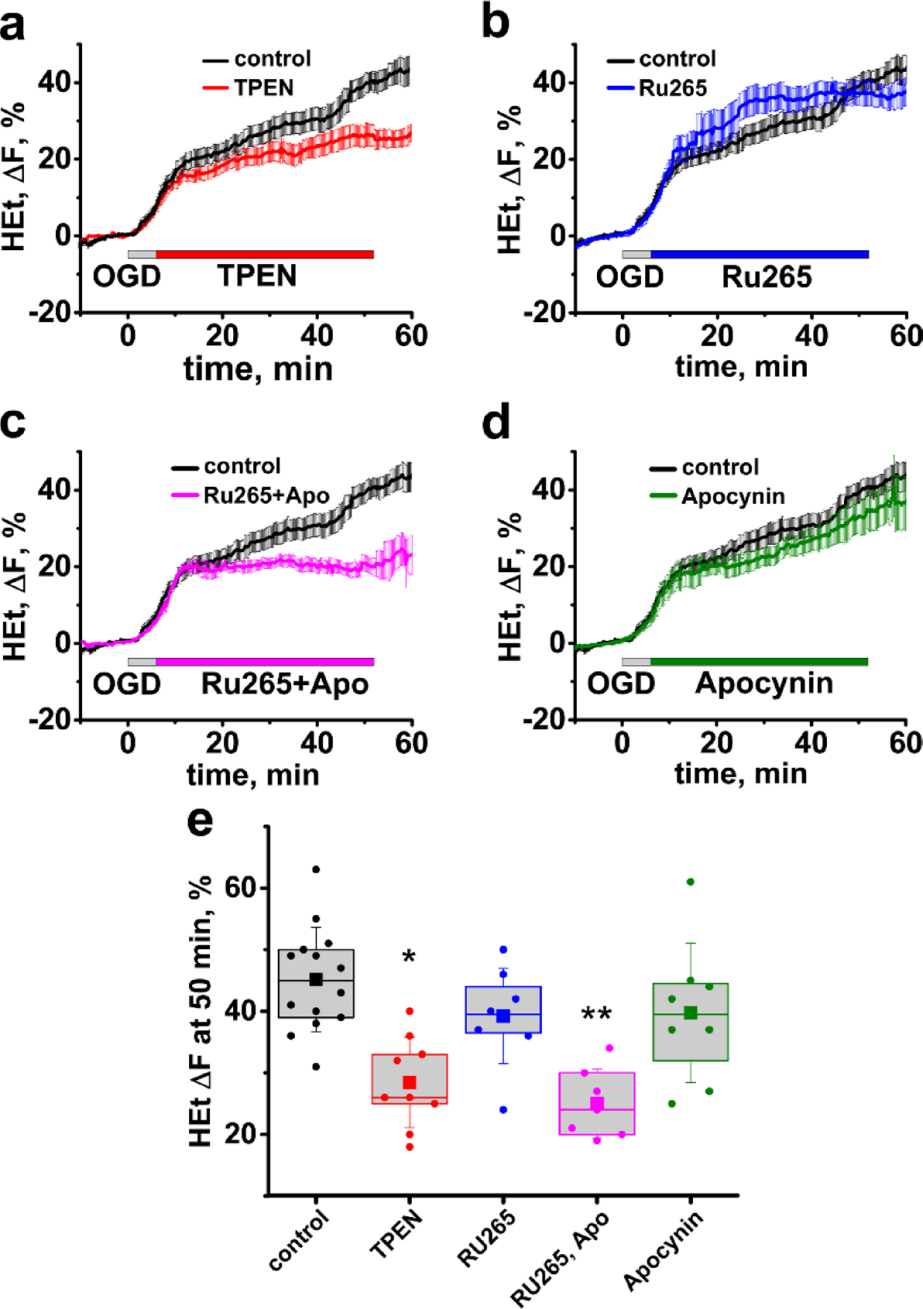
Mitochondrial Ca^2+^ and Zn^2+^ accumulation through the MCU contribute to the post-ischemic ROS overproduction. (**a**–**d**) Average traces show changes in HEt fluorescence in control slices subjected to OGD alone (black trace) and in experiments where treatments were administered *after OGD withdrawal*, as indicated. (**a**) Zn^2+^ chelation with TPEN after OGD (red trace) attenuates the sharp increase in ROS generation. (**b**) MCU inhibition with RU265 changes dynamics but does not decrease ROS production (blue trace). (**c**) MCU blockade combined with inhibition of NOX by apocynin considerably diminishes the reperfusion-evoked ROS burst (magenta trace). (**d**) NOX inhibition alone (olive) does not notably affect ROS. (**e**) Box chart shows average HEt ΔF 50 min after the start of OGD. *p = 0.02, **p < 0.01 vs control.

Since our previous studies found substantial and long-lasting Zn^2+^ accumulation in CA1 mitochondria after reperfusion, and also found that Zn^2+^ is a potent contributor to ROS generation by neuronal mitochondria in culture, we questioned whether the Zn^2+^ contributes to the reperfusion-evoked ROS overproduction. Indeed, we found that Zn^2+^ chelation with TPEN applied after OGD termination decreased the late surge in ROS after reperfusion (HEt ΔF = 25.7 ± 2.6%, n = 10 with TPEN vs 39.7 ± 2.8%, n = 14 slices in control, OGD alone, *p* = 0.02, evaluated 50 min after OGD onset, Fig. 4a,e).

Our past studies found that by ~ 30 min after OGD Zn^2+^ is cleared from cytoplasm and accumulates in mitochondria, where it stays for prolonged periods of at least 1 h^26,35^. Thus, we sought to assess whether post-ischemic ROS generation depends specifically upon Zn^2+^ entrance to mitochondria. To prevent Zn^2+^ accumulation to mitochondria we blocked the MCU after OGD withdrawal using the recently developed MCU inhibitor Ru265^36^. Surprisingly, while MCU inhibition changed the dynamics of ROS generation, it also caused an increase in ROS production during first ~ 20 min after OGD, thus having little net effect on the total amount of ROS 50 min after the start of OGD (HEt ΔF = 37.7 ± 3.2%, n = 7 with Ru265, vs 39.7 ± 2.8%, n = 14 in control, *p* = 1, Fig. 4b,e). We hypothesized that since MCU inhibition prevents both Zn^2+^ and Ca^2+^ entrance into mitochondria, it likely causes a greater Ca^2+^ accumulation in the cytoplasm, which can lead to overactivation of NOX—another possible source of ROS after ischemia^5,37,38^. Indeed, co-treatment with Ru265 and the NOX inhibitor apocynin largely eliminated the post-ischemic ROS burst (HEt ΔF = 25 ± 2.1, n = 7, vs 39.7 ± 2.8%, n = 14 in control, *p* < 0.01, evaluated 50 min after the start of OGD, Fig. 4c,e). Treatment with apocynin alone had a small, non-significant effect on ROS production during reperfusion (HEt ΔF = 32.3 ± 3.7%, n = 8, *p* = 0.13 compared to control, Fig. 4d,e).

## Discussion

While prolonged global ischemia results in immediate brain death, with shorter ischemic episodes neurons often initially recover. However, many sensitive neurons, particularly CA1 hippocampal pyramidal neurons, die within hours to days later. Thus, many deleterious events leading to neuronal death occur after reperfusion, a time when blood flow to the brain is restored and interventions are possible. The role of ROS in reperfusion-associated neuronal injury has long been established by many studies that documented oxidative damage to multiple types of intracellular molecules^15,17,39^. Nonetheless, the specific mechanisms and, thus, targets for interventions are largely not understood, and most of our knowledge about the dynamics of ROS production during and after ischemia originates from experiments utilizing dissociated cultures^5^.

Our study used short transient OGD and reperfusion in acute hippocampal slices loaded with the ROS sensitive indicator, HEt, to monitor changes in ROS generation. Slices comprise native brain tissue and connectivity and recapitulate many of the events occurring during in vivo ischemia while permitting precise environmental control and monitoring of responses. We observed a two-phase acceleration of ROS generation: the first one starts during OGD and continues into early reperfusion, and the second phase is a delayed burst of ROS occurring ~ 40 min after OGD termination. We found no prior observations of this “late” burst and, more importantly, barely any information related to possible mechanisms involved in post-ischemic ROS overproduction. As possibilities for intervention are very limited during ischemia, when the first acceleration of ROS production occurs, we investigated the factors contributing to the second, delayed, ROS burst.

As we hypothesized that OGD/reperfusion evoked Ca^2+^ accumulation in mitochondria causes overactivation of the ETC, leading to mitochondrial hyperpolarization and excessive ROS generation, we investigated whether mitochondrial hyperpolarization indeed occurs after reperfusion and contributes to the ROS burst. We found distinct hyperpolarization of ΔΨ_m_ ~ 30 min after OGD withdrawal. We further found that treating slices after OGD termination with either low-dose FCCP or FTY720, both of which we found to cause a mild (~ 10%) loss of ∆Ψ_m_, largely suppressed the delayed ROS burst—supporting the idea that mitochondrial hyperpolarization after reperfusion is an important contributor to the delayed ROS production.

We also found that Zn^2+^ chelation with TPEN inhibits the late ROS burst, supporting the critical role of Zn^2+^ in post-ischemic ROS production. As our previous studies discovered that after ischemia Zn^2+^ quickly accumulates in CA1 mitochondria^26^, we suggest that Zn^2+^-mediated ROS acceleration after OGD also depend upon Zn^2+^ accumulation to mitochondria. Thus, we assessed whether inhibiting Zn^2+^ entrance to mitochondrial affects ROS generation. Surprisingly, while blocking the MCU attenuated the delayed ROS burst, it appeared to result in accelerated ROS generation at earlier time points after reperfusion (Fig. 4b). We questioned whether this earlier increase in ROS might be because MCU blockade prevents mitochondrial Ca^2+^ buffering resulting in an increased cytosolic Ca^2+^ load which could trigger ROS generation by NOX activation^40^. Indeed, combined application of the MCU inhibitor RU265 with the NOX antagonist, apocynin, strongly attenuated the ROS generation. Apocynin by itself had little effect on the observed delayed ROS burst, suggesting that NOX makes a relatively small contribution to the total ROS production in the absence of MCU blockade.

### Mitochondrial Ca^2+^ accumulation is a trigger of reperfusion-associated mitochondrial hyperpolarization and ROS overproduction

“Excitotoxicity” has long been implicated as a major contributor to ischemic injury. Critical early studies demonstrated that Ca^2+^ entrance through NMDA-activated channels contributes to neuronal injury in many pathological conditions including ischemia^41–43^. However, clinical trials targeting NMDA receptors failed to result in any substantial protection after ischemia^44,45^. This can be explained by the fact that most of the Ca^2+^ accumulation occurs during the acute ischemic event when there is little opportunity for intervention. A more beneficial approach would be to target downstream events following ischemic Ca^2+^ accumulation.

Multiple studies found that during ischemia Ca^2+^ quickly accumulates in mitochondria^12–14^, and mitochondria were proposed to be important targets for deleterious effects of Ca^2+^^10,11,46^. It has been shown that elevated Ca^2+^ is a potent activator of the mitochondrial respiratory chain causing increased respiration and ROS production^9,16,47–49^. This mechanism likely serves as an adaptation to increase ATP production during physiological conditions of high energy demand^46^. However, during pathological ischemic conditions, mitochondrial Ca^2+^ overload probably causes the respiratory chain overactivation but without oxidative phosphorylation and ATP production due to lack of oxygen. Consequently, after reperfusion (upon restoration of oxygen and glucose) overactivation of the ETC likely leads to enhanced and prolonged hyperpolarization of ΔΨ_m_. Sanderson et al.^9^ in their comprehensive review proposed that this mitochondrial hyperpolarization contributes to ROS overproduction after ischemia. The idea of the deleterious role of reperfusion-related mitochondrial hyperpolarization was further supported by experiments manipulating the mitochondrial uncoupling protein 2 (UCP2). Deletion of UCP2 increased ROS production and exacerbated brain injury after middle cerebral artery occlusion in mice^50^, while UCP2 overexpression protected the brain after cerebral ischemia^51^. Furthermore, several studies found that mitochondrial hyperpolarization causes an exponential increase in ROS production^18,19,52^.

### Mitochondrial Zn^2+^ accumulation after reperfusion contributes to ROS overproduction

Our previous studies found that long-lasting Zn^2+^ accumulation occurs after ischemia in the mitochondria of highly vulnerable CA1 neurons, but not in CA3 neurons that are more resistant to ischemic injury. Moreover, we also found that this Zn^2+^ accumulation contributes to delayed mitochondrial damage^25,26^. The mechanism of Zn^2+^-associated mitochondrial injury was not clear, but studies using cultured neurons and isolated mitochondria found that mitochondrial Zn^2+^ accumulation is an important contributor to ROS generation^23,27–29^. One of the possible mechanisms is that Zn^2+^ has been shown to cause irreversible inhibition of mitochondrial enzymes of antioxidant defense^30^. A feed-forward process is also likely involved since ROS, in turn, causes more Zn^2+^ to be released from Zn^2+^-binding proteins such as metallothionein-III resulting in increased mitochondrial Zn^2+^ loading^22,53^. Furthermore, Zn^2+^ is able to stay in the matrix for hours, likely exacerbating mitochondrial damage^35^. Conversely, we did not find substantial delayed Ca^2+^ accumulation in mitochondria at 30 min and 1 h after ischemia, likely reflecting mitochondrial ability to clear Ca^2+^ relatively quickly via the Na^+^/Ca^2+^ exchanger, which is the main mediator of Ca^2+^ release from mitochondria in neurons^26,54–56^. In conclusion, our observations of long-lasting Zn^2+^ accumulation in mitochondria after reperfusion and decrease of post-ischemic ROS production by either Zn^2+^ chelation or prevention of Zn^2+^ entrance to mitochondria support a key contribution of mitochondrial Zn^2+^ accumulation after ischemia to oxidative damage of CA1 neurons.

### NOX and ischemic ROS generation

NOX was recently found to be the main source of ROS production after NMDA exposure to cultured neurons^40^. Consistent with this result, while we did not find NOX activation to be a major contributor to reperfusion-evoked ROS generation, our data suggest that NOX is involved, particularly if cytosolic Ca^2+^ is elevated due to block of mitochondrial Ca^2+^ uptake via MCU blockade; thus, targeting NOX in combination with other treatments (like MCU blockade) might be beneficial.

### Therapeutic implications

Therapies for ischemic brain injury are extremely limited for several reasons including: 1) limited knowledge of critical events leading to neuronal injury and death, and 2) inability to deliver drugs to sites of ischemic injury until blood flow is restored. Consequently, the primary focus to date has been to restore blood flow as quickly as possible. However, processes set into motion by even relatively brief (several minute) episodes of ischemia can lead to delayed neuronal injury and death. Oxidative damage due to ROS production has long been felt to be a major contributor to the post-ischemic injury.

**Fig. 5:**
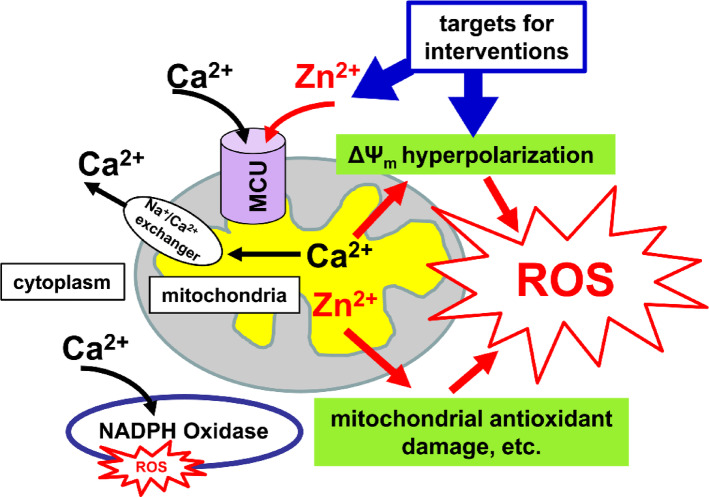
Events likely occurring after ischemia and contributing to ROS production. Mitochondria repolarize/hyperpolarize and commence reuptake of Zn^2+^ and Ca^2+^ (These ions initially enter mitochondria during ischemia and are released into cytosol upon loss of ∆Ψ_m_) Ca^2+^ is then released from mitochondria through the Na^+^/Ca^2+^ exchanger, but Ca^2+^ triggered overactivation of the ETC and consequent mitochondrial hyperpolarization causes accelerated ROS production. In the cytoplasm Ca^2+^ can activate NOX, which causes additional ROS generation. Zn^2+^ stays in mitochondria for a prolonged period of time, causing mitochondrial dysfunction and contributing to the delayed ROS burst. The mitochondrial hyperpolarization and the Zn^2+^ accumulation in mitochondria (possibly in combination with NOX inhibition) can be targeted after restoration of blood flow to prevent excessive ROS generation.

Our studies revealed a major burst of mitochondrial ROS production appearing after a delay (~ 40 min) following an episode of acute ischemia. This delay before maximal ROS production may permit delivery of drugs when blood flow is restored after the acute ischemic episode. Present findings suggest 2 mechanisms that appear to contribute importantly to the delayed mitochondrial ROS generation after transient ischemia: hyperpolarization of the mitochondria, to which Ca^2+^ accumulation in mitochondria through the MCU likely contributes, and Zn^2+^ accumulation in mitochondria through the MCU possibly damaging the mitochondria antioxidant system (Fig. 5). Targeting these mechanisms with drugs delivered after reperfusion may provide a valuable new approach for interrupting the pathogenic cascade, thereby resulting in better treatment outcomes.

### Study limitations

A limitation of the study is the absence of ratiometric ROS indicators that can be reliably used in slices. We found that hydroethidine is the most suitable for detecting changes in ROS production in brain slices both at baseline and during ischemia/reperfusion. However as it is non-ratiometric, its fluorescence is affected by short-lasting but substantial cell swelling evoked by spreading depolarization that usually occurs between 6 and 8 min after the start of OGD, and also by slowly developing cell swelling starting at some point after 1 h of reperfusion. This limits both the duration of OGD and time of recording after OGD termination. Additionally, it is possible that slight swelling which can occur in some slices even after 6 min OGD might mask some of the ROS production increase, resulting in underestimations of actual ROS generation changes.

## Data Availability

The datasets used and/or analyzed during the current study are available from the corresponding author on reasonable request.
